# Transcriptional regulation of astrocyte heterogeneity and disease pathogenesis across six different neurodegenerative diseases and seven brain regions

**DOI:** 10.1002/alz70855_098860

**Published:** 2025-12-23

**Authors:** Ibrahim Olabayode Saliu, Wei Feng, Shinnosuke Yamada, Qingyun Li, Ching On Wong, Erik S. Musiek, Guoyan Zhao

**Affiliations:** ^1^ Washington University School of Medicine, St. Louis, MO, USA; ^2^ Washington University, St. Louis, MO, USA; ^3^ The State University of New Jersey, Newark, NY, USA; ^4^ Knight Alzheimer Disease Research Center, Saint Louis, MO, USA; ^5^ Washington University School of Medicine, Saint Louis, MO, USA; ^6^ Washington University School of Medicine, St Louis, MO, USA

## Abstract

**Background:**

snRNA‐seq has advanced our understanding of astrocyte heterogeneity. However, studies vary in biological factors (disease conditions and brain regions), technical approaches, and analytical methods, making it challenging to distinguish whether observed astrocyte heterogeneity reflects true biological differences or technical variations. Currently, the number of astrocyte subpopulations in different brain regions, the similarities and differences of astrocyte and their responses to diseases remain poorly understood.

**Method:**

We conducted uniform analysis of eight human snRNA‐seq datasets using the same analytical approaches and parameters. The datasets were derived from seven brain regions and six neurodegenerative diseases (NDs) including Alzheimer's disease, Parkinson's disease, Parkinson disease dementia, dementia with Lewy bodies, Huntington disease, and Nasu‐Hakola disease. We compared astrocyte heterogeneity, transcriptional regulation, and disease‐associated transcriptomic changes.

**Result:**

We identified three astrocyte subpopulations: Ast‐0 and Ast‐2 were present across all eight datasets, while Ast‐1 was found in six datasets. This identification of homologous populations enabled comparative analysis of astrocyte reactivity, disease response, and transcriptional control across diseases and brain regions. We defined core conserved marker genes and pathways shared across all conditions for each astrocyte subpopulation. Notably, Ast‐1 across all datasets showed unique enrichment for amyloid‐beta binding and ND‐related pathways suggesting a specific role of Ast‐1 in neurodegeneration. Differentially expressed genes (DEGs) in disease conditions were largely shared among astrocyte subpopulations within each disease and brain region, indicating coordinated responses to disease pathology. Importantly, both canonical and non‐canonical Wnt signaling pathways along with their downstream targets, and endolysosomal pathways were concordantly dysregulated across all disease conditions and brain regions. This suggests these pathways may represent a common mechanism underlying ND pathogenesis. By ranking disease DEGs based on their frequency of dysregulation across datasets, we identified both known disease risk genes and novel candidates. We defined consensus transcription regulatory networks controlling astrocyte subpopulation identities. Functional validation through astrocyte‐specific knockdown of PBX1 and SOX2 resulted in endocytosis defects in primary mouse astrocytes and motor defects in flies, confirming their astrocyte‐specific functions.

**Conclusion:**

Our analysis revealed astrocyte‐specific molecular changes shared across six NDs and seven brain regions. These findings illuminate the common mechanism underlying different dementias and provide potential novel therapeutic targets.

